# Effect of thermal and non-thermal processing on Technofunctional, nutritional, safety and sensorial attributes of potato powder

**DOI:** 10.1016/j.fochx.2024.101896

**Published:** 2024-10-16

**Authors:** Muhammad Waseem, Saeed Akhtar, Tariq Ismail, Tawfiq Alsulami, Muhammad Qamar, Dur-e-shahwar Sattar, Raheel Suleman, Wisha Saeed, Crossby Osei Tutu

**Affiliations:** a Department of Food Science & Technology, Faculty of Agriculture & Environment, Islamia University Bahawalpur, Bahawalpur 63100, Pakistan; b Department of Food Science & Technology, Faculty of Food Science & Nutrition, Bahauddin Zakariya University, Multan 60800, Pakistan; c Department of Food Science & Nutrition, College of Food and Agricultural Sciences, King Saud University, Riyadh 11451, Saudi Arabia; d Department of Family and Consumer Sciences, University of Ghana, Accra, Ghana

**Keywords:** *Solanum tuberosum*, Processing, Value addition, Microwave, Pesticides, Antinutrients

## Abstract

Potato is a highly nutritious staple food however, it also contains some antinutrients like alkaloids, phytates, tannins, oxalates as well as pesticide residues. Therefore, this study was conducted to reduce the loads of antinutrients and pesticides in potato powder (PP) using thermal and non-thermal processing techniques. Nutritional analysis revealed that the raw PP contained significantly (*p* < 0.05) higher magnitudes of dietary proteins (10.2 %), fibers (6.3 %), Na (50 mg/100 g), Ca (62 mg/100 g) and K (988 mg/100 g) when compared with the processed PP. The results demonstrated that all thermal and non-thermal processing techniques significantly reduced the antinutrients and pesticide residues. However, microwave heat treatment anticipated the highest reduction in alkaloids, oxalates, tannins and phytates contents from 60 to 14 mg/100 g (76 % reduction), 31–6 mg/100 g (80 % reduction), 91–15 mg/100 g (84 % reduction) and 45–8 mg/100 g (82 % reduction), respectively. Additionally, microwave heat processing also exhibited the highest decline in imidacloprid, cypermethrin, bifenthrin, chlorpyrifos and deltamethrin contents by 87 %, 76 %, 63 %, 79 % and 81 %, respectively. Later, microwave-treated PP (the most effective treatment) was used to develop unleavened flatbreads (i.e., chapatis) @ 2–10 %. Organoleptic evaluation of supplemented flatbreads suggested that 5 % supplementation with microwave treated PP has the highest overall acceptability. Therefore, it is concluded that thermal and non-thermal processing techniques are effective tools to reduce loads of antinutrients and pesticide burden in potatoes. Moreover, the study also suggests, PP can be efficiently used as natural food supplement for development of value-added foods.

## Highlights

• Potato powder (PP) upholds higher magnitudes of fibers, proteins, Ca, Zn & Fe.
• Thermal & non-thermal processing declined pesticides & antinutrients in potato powder
• Addition of processed PP in flatbreads resulted in higher consumer acceptability.
• Based on technofunctional attributes PP can be used in soups, gravies & curries.

## 1 Introduction

Potato (*Solanum tuberosum* L.), a nutrient enriched food crop which belongs to family Solanaceae, is ranked at fourth after the wheat, rice and maize (Guo et al., 2023). With a global cultivation area of 17.3 MH, production statistics reveal global production of potato is about 359 million tons, while for Pakistan it is recorded as 5,872,960 tons (FAOSTAT, 2021). The leading potato producing regions around the globe include People's Republic of China, Indo-Pak region, Ukraine and Russia (Waseem, Akhtar, Ahmad, et al., 2022). Potatoes have gained global attention due to attractive nutritional profile as the tuber contain significant magnitudes of dietary fibers, essential proteins (i.e., essential amino acids), vitamins (B_1_, B_6_, B_9_, C, E and K) and minerals (Mn, Na, Ca, P, K and Mg) (Akonor et al., 2023; Franková et al., 2022; Mi et al., 2022; Akonor et al., 2022)*.* Potato proteins are quite comparable with cereals and egg proteins. Therapeutic properties of potatoes are known to be linked with its health promoting phytonutrients such as anthocyanins (malvidin, peonidin, delphinidin, cyanidin and petunidin), polyphenols (flavonoids), carotenoids and patatin protein (Kita et al., 2013; Sun et al., 2021; Osei Tutu et al., 2024), which exhibit crucial role in the management of numerous health disorders such as diabetes, cardiovascular disorders, inflammation, obesity, hyperlipidemia, hypertension, allergy, neurodegenerative problems, cancers and viral infections (Kowalczewski et al., 2022; Yang et al., 2023; Osei Tutu et al., 2023). Higher water absorption and swelling capacities and better starch digestibility, potatoes are viable ingredients are supposed as viable choice in a wide array of food applications. Modes of dietary uses of potatoes are dehydrated powders for gravies, soups, cakes, bread, noodles, curries, sausages, beverages, snacks and gluten-free cookies (Osei Tutu et al., 2024; Akhobakoh et al., 2022; Kowalczewski et al., 2022; Whitney & Simsek, 2020).

Plant origin antinutrients such as tannins, phytic acids, oxalates, enzyme inhibitors and saponins interfere with the minerals and adversely affect their bioavailability and absorption(Akonor et al., 2023). These intrinsic toxicants are of serious concern where legumes, cereals, pulses and fresh vegetables are consumed as staple diets, which however; may cause deleterious health effects like micronutrient inadequacies, kidney stones, hepatic issues and neuro problems (Faizal et al., 2023). Pesticides are abundantly used in fruits and vegetables as sustainable means of controlling insects, pests and weeds to overcome the ever-rising plant diseases (Tang et al., 2023). According to recent global statistics, the use of pesticides has exceeded over 4.1 million tons per year, of which herbicides reveal about 71.4 % use, followed by fungicides, insecticides and rodenticides by 10.4 %, 18.2 % and 17 %, respectively (Centanni et al., 2023). Indiscriminative use and over dependence on pesticides to overcome agricultural insects and pests' leads to the accumulation of pesticides in edible food crops, which consequently affects neuro functionalities, reproductive organs, liver functioning, kidney, respiratory system and cancer (Rani et al., 2021). The maximum residue limits (MRL's) of different pesticides including imidacloprid, cypermethrin, bifenthrin, chlorpyrifos and deltamethrin for potatoes have been documented by FAO/WHO, Codex Alimentarius Commission range between 0.01 and 2 ppm (CAC, 2011). Potato tubers are among the vegetables which are considered among the most susceptible food crops towards the attack of insects and pests. In the same sense, potatoes have been listed among the ‘dirty dozen’ fruits and vegetables holding the highest levels of pesticides (Nguyen et al., 2020). Earlier studies report the presence of pyrethroids (deltamethrin and cypermethrin) and organochlorines (*γ*-chlordane, lindane, heptachlor and dimethachlor) in potatoes beyond their allowed MRL levels (Terfe et al., 2023). Globally, indiscriminate use, weaker legislative controls and dietary exposure to pesticides have raised a number of health challenges in humans (Terfe et al., 2023), including kidney failure, neurotoxicity, obesity, bronchitis, musculoskeletal abnormalities, bronchitis, infertility problems, congenital malformations, endocrine alterations, neurodegenerative issues, neuropsychiatric disorders, genotoxicity, behavioral disorders, liver dysfunction and cancer (Cancino et al., 2023; Panis et al., 2022).

In recent years, scientists have explored new approaches like thermal and non-thermal processing to mitigate the ever increasing burden of intrinsic and extrinsic toxicants in fruits and vegetables to improve their nutritional quality (Waseem, Akhtar, Qamar, et al., 2022). Retrospective studies are evident of employing the thermal, non-thermal, physical, chemical and biological food processing techniques including blanching, boiling, acid soaking, saline soaking, alkaline soaking, freezing, fermentation, frying and drying (Acheampong et al., 2024; Faizal et al., 2023; Tang et al., 2023), peeling, washing, baking and juicing (Nguyen et al., 2020; Terfe et al., 2023) as viable, cost-effective and labor-less techniques to reduce the pesticides and antinutrients burden in vegetables. Recent studies have indicated that microwave heat processing and autoclaving as the most effective thermal techniques in declining antinutrients in vegetables typically attributed to the leaching and rupturing of plant cell walls (Natesh et al., 2017; Samtiya et al., 2020). Available literature have endorsed the great potential of microwave heat processing and blanching as viable techniques in mitigating the loads of antinutrients in vegetables when compared with the various processing methods like soaking and resulted in decline of about 90 % of the antinutrients and pesticides and improved the safety of vegetables for the human consumption (Javed et al., 2024). Likewise, earlier studies have also validated the use of blanching, microwave heating, acid soaking and base soaking to exert significant reduction in intrinsic (antinutrients like phytates, tannins, oxalates and alkaloids) and extrinsic toxicants (i.e., pesticide residues) between 80 and 92 % in vegetables (Waseem et al., 2024).

Plethora of useful information is available on nutritional and anti-nutrient indices of raw potatoes and its food uses. However, earlier studies have identified a gap in the availability of a comparison of detoxification techniques for the efficient detoxification of potatoes and their effects on the physicochemical and technofunctional characteristics of the processed versions of potatoes. Therefore, this study was strategized to investigate the impact of thermal and non-thermal processing to detoxify antinutrients and pesticide residues in potatoes to set maximum allowable limits and to utilize the detoxified PP in the development of unleavened flatbread with better sensory acceptability.

## 2 Materials and methods

### 2.1 Procurement of raw materials, chemicals and reagents

Fifteen kilograms of disease, insect and pest-free fresh potatoes were purchased from Dua Enterprises, Agro-Farm, Multan, Pakistan. The potatoes were manually washed with clean drinking water, peeled with a knife, sorted and graded for quality and sliced with a household slicer in the laboratory of the Department of Food Science & Technology, Faculty of Food Science and Nutrition, Bahauddin Zakariya University, Multan, Pakistan. Reagents and chemicals used in nutritional, technofunctional and physicochemical trials were all analytical grade and procured from local distributors of Sigma Chemical Co., Ltd. (St. Louise, MO) and Merck (Darmstadt, Germany). Standards used in the determination of antinutrients including alkaloids, oxalates, tannins and phytates, minerals (Ca, Na, K, Fe and Zn) and pesticide residues (cypermethrin, imidacloprid, bifenthrin, deltamethrin and chlorpyriphos) were purchased from BDH Chemicals Ltd. (Shanghai, China).

### 2.2 Processing treatments of potatoes

#### 2.2.1 Microwave heat processing and blanching

After preliminary operations potatoes were microwave heat processed at 1.1 kW for 2 min (Waseem, Akhtar, Ahmad, et al., 2022), blanched in clean distilled water at 100 ± 2 °C for 1.5 min. On completion of blanching, the slices were removed from distilled water and pre-dried using absorbent paper.

#### 2.2.2 Acid and alkali soaking

Uniformly sized potatoes were soaked in 2 M hydrochloric acid (HCl) for acid soaking. Contrarily, the slices were soaked in alkaline solutions consisting of 1 % sodium bicarbonate and, a mixture of salt solutions (i.e., 1.5 % and 0.5 % of sodium bicarbonate and sodium carbonate) for a period of 16–18 h (Waseem, Akhtar, Qamar, et al., 2022).

### 2.3 Raw and processed PP preparation

The protocol of (Akter et al., 2023) was followed to prepare powders from raw and processed potatoes. Uniformly sliced pieces of raw and processed potatoes were evenly laid over the nylon mesh (dimension = 0.186 × 0.186 m^2^) for cabinet drying (Pamico Tech, Faisalabad, Pakistan) at 45 ± 2 °C, 12–14 h to a moisture content of 8–10 %. Thereafter, dehydrated slices were converted into fine powders (mesh size = 80 mm) using heavy-duty grinder (Pamico Tech, Faisalabad, Pakistan). PP were stored in airtight glass jars at 4–6 ± 2 °C for further analyses.

### 2.4 Functional properties of raw and processed PP

#### 2.4.1 Bulk density

Bulk densities of PP were estimated in accordance with the protocol as documented by (Buzera et al., 2022). Ten grams of each powder was filled into a 100 mL graduated cylinder and final bulk volume (V) was measured. Bulk density was calculated as:

(I) $$\text{Bulk density}\;\left(\mathrm{BD}\right)=\frac{\text{Mass}\;\left(g\right)}{\text{Volume}\;\left(\mathrm{mL}\right)}$$

#### 2.4.2 Rehydration ratio (RR)

PP rehydration ratios were determined as outlined by (Okonkwo et al., 2022). Accurately measured five-gram sample was mixed in 50 mL distilled water for 30 min. Subsequently, filtration was performed using Whatman filter no. 41. Permeates of each sample were weighed and RR was estimated as:

(II) $$\text{Rehydration ratio}=\frac{\text{Rehydrated Sample Weight}\;\left(g\right)}{\text{Dehydrated Sample Weight}\;\left(g\right)}$$

#### 2.4.3 Water solubility index (WSI) and water absorption index (WAI)

WSI and WAI of dehydrated powders were measured in accordance with the protocols as measured by (Castro-Mendoza et al., 2022). Accurately weighed one gram of sample was added into 10 mL distilled water and mixed for 30 min. Subsequently, the mixture was centrifuged (Hermle Z236K, Wehingen, Germany) at 4300 rpm for 20 min. The supernatant was collected in a pre-weighed dish and evaporated using hot air oven (Memmert UNB 200, Munich, Germany) at 105 ± 2 °C for 8 h to obtain constant weight. The results for water solubility index and water absorption index were determined using the following eqs. (III), (IV):

(III) $$\mathrm{WSI}\;\left(\%\right)=\frac{\text{Dehydrated Supernatant weight}\;\left(g\right)}{\text{Initial weight of dehydrated sample}\;\left(g\right)}\times 100$$

(IV) $$\mathrm{WAI}\;\left(g\;\text{water}/g\;\text{sample}\right)=\frac{\text{Weight of tube with sediment}\;\left(g\right)-\text{Weight of tube with sample}\;\left(g\right)}{\text{Initial weight of sample}\;\left(g\right)}\;$$

#### 2.4.4 Swelling power

(Moreno-Ochoa et al., 2023) method was followed to determine the swelling power of each sample. Three grams of each sample was mixed in 30 mL of distilled water in a pre-weighed falcon tube (i.e., 50 mL) to make powder-water slurry and heated in water bath (WNB-29, Memmert, Schwabach, Germany) at 60 °C for 30 min with constant stirring. Falcon tubes containing hot slurry were centrifuged at 1500 rpm for 10 min. Subsequently, supernatants and solid residues were collected and weighed in pre-weighed dishes and falcon tubes, respectively. Swelling powers of all samples were calculated by using equation mentioned hereunder (V):

(V) $$\mathrm{Sp}\;\left(g\;\text{paste}/g\;\mathrm{dry}\;\text{sample}\;\right)=\frac{\text{Weight of solid paste}\;\left(g\right)}{\text{Weight of sample}\;\left(g\right)}$$

#### 2.4.5 Hygroscopicity

Hygroscopicity of dehydrated powders were determined as laid down by (Arebo et al., 2023). Two gram of powder sample was kept in *desiccator* at 25 °C already containing saturated NaCl solution at 70 % relative humidity. After one week, each sample was measured again for weight and hygroscopicity values were expressed as given below.

(VI) $$\text{Hygroscopicity}\;\left(\%\right)=\frac{\text{Gain in weight of sample}\;\left(g\right)}{\text{Original weight of sample}\;\left(g\right)}\times 100$$

### 2.5 Nutritional composition

Nutritional composition consisting of moisture (No. 925.10), crude ash (No. 923.03), fat (No. 920.85), fiber (No. 32–10) and protein (No. 920.87) contents of raw and processed PP and supplemented unleavened flatbread were estimated as mentioned in standardized methods of Official Methods of Analysis (Latimer, 2019). Whereas carbohydrates and calorie contents were calculated as;

(VII) $$\text{Carbohydrates}\;\left(g/100\;g\right)=\left[100-\left(\text{moisture}+\text{crude}\;\mathrm{ash}+\text{crude}\;\mathrm{fat}+\text{crude fiber}+\text{crude protein}\right)\right]$$

(VIII) $$\text{Calories}\;\left(\text{kcal}/g\right)=4\;\left(\text{Protein}\%+\text{Carbohydrates}\%\right)+9\;\left(\mathrm{Fat}\%\right)$$

However, mineral elements consisting of Ca, K, Na, Fe and Zn in powder samples were estimated by following the method as mentioned by (Raletsena et al., 2023).

### 2.6 Antinutrients

#### 2.6.1 Determination of alkaloids

Dehydrated PP were assessed for alkaloid contents as described by (Khan et al., 2019). Five grams of each sample was amalgamated in 10 % ethanol-acetic acid solution (i.e., 100 mL) and were given stay for 4 h and filtered via Whatman filter no. 41 and filtrate was concentrated using water bath. Afterwards, filtrate was mixed with concentrated ammonium hydroxide (NH_4_OH) to form alkaloid precipitates. The residues were given second filtration and washing using 1 % ammonium hydroxide. Finally, the mixture was oven dried at 60 °C for 30 min to record the alkaloid contents as:

(IX) $$\text{Alkaloids}\;\left(\%\right)=\frac{\text{Weight of precipitates}\;\mathrm{wih}\;\text{filter}-\text{Weight of empty filter}\;\left(g\right)}{\text{Original weight of sample}\;\left(g\right)}\times 100$$

#### 2.6.2 Determination of oxalates

Two grams of each sample was digested in 190 mL distilled water and 10 mL, 6 M hydrochloric acid in water bath. After digestion, centrifugation was performed at 2000 rpm for 10 min and was concentrated on hot plate to adjust the final volume up to 25 mL. Thereafter, filtration was performed via Whatman filter paper no. 41 to collect the brown colored precipitates. Then, three drops of methyl indicator were added followed by the addition of concentrated ammonia to obtain faint yellowish appearance. Subsequently, 10 mL of 5 % calcium chloride was added with constant stirring to precipitate the oxalates and final filtration was performed. Filtrates were titrated against 0.5 % potassium permanganate until pink colored end point not persisted for 30 s (Abdullahi et al., 2021). Oxalates were measured by formula as mentioned below.

(X) $$\text{Oxalates}\%={}^{*}\text{Titer value}\times 0.1125$$

(XI) $${}^{*}1\;\mathrm{mL}\;\text{of potassium permanganate}=2.24\;\mathrm{mg}\;\text{of oxalates}$$

#### 2.6.3 Determination of tannins

(Khan et al., 2019) protocol was adopted to assess tannin contents in dehydrated powders. Accurately measured 0.5 g of each sample was mixed with 50 mL of distilled water and stirred for 1 h. Thereafter, reaction mixture was filtered and 5 mL supernatant was taken to mix with 2 mL, 0.1 M ferric chloride in a test tube. The absorbance of each sample was measured in 10 min at 395 nm on spectrophotometer (UV–Vis 3000, Darmstadt, Germany). Final concentrations of tannins (mg/100 g) were computed against tannic acid standard curves.

#### 2.6.4 Determination of phytates

(Sharma et al., 2022) method was followed to estimate phytates in PP. About 0.5 g of each sample was digested in 0.2 N hydrochloric acid on hot plate (LMS-1003, Daihan Labtech Co. Ltd., Namyangju, South Korea) for 30 min with persistent stirring and then allowed to stay for 10 min. Afterward, 0.5 mL of each extract was taken in test tube and covered with stopper. Thereafter, one milliliter ferric solution (Conc. i.e., 0.2 g ammonium iron III sulphate mixed with 2 N, HCL reaching to a final volume of 1 L) was added to reaction mixture and heated in water bath for 30 min and cooled to room temperature using ice water. Subsequently, the mixture was centrifuged for 30 min at 3000 rpm and 1 mL of supernatant was thoroughly mixed with 2 mL of 2,2′-bipyridine solution (Conc. i.e., 10 g of 2,2′-bipyridine and 10 mL of thioglycolic acid dissolved to a total volume of 25 mL in distilled water). Sodium phytate-phosphorous standards were prepared for development of standard curves. Absorbance reading of standard, reagent blank and sample was taken at 519 nm using UV–Vis Spectrophotometer. The results for phytates contents were expressed as mg/100 g of phytates.

### 2.7 Pesticide residues extraction, cleanup and determination

Precisely measured 50 g homogeneous sample of each powder was mixed with 20 g of anhydrous sodium sulfate in 250 mL Erlenmeyer flask. Then, 10 mL saturated sodium chloride and 70 mL ethyl acetate in were added in it and gently mixed at 50 rpm for 1 h in orbital shaker (MaxQ4000, Thermo Scientific TM, Waltham, MA, USA) and filtered using Whatman filter paper no. 1. Filtered samples were stored at 2–4 ± 2 °C in plastic vials. Now, the extracts were passed through anhydrous sodium sulfate column followed by activated charcoal and silica gel 7:5 (*w*/w) columns adjusted @ 1 mL/min flow rate for cleanup of any color residues. Afterwards, the contents were also eluted for 2nd cleanup using 50 mL of methanol: acetone (7:3 *v*/v) solution. The eluent was collected in plastic vials and evaporated to a final volume of 1–2 mL using rotary evaporator at 45 °C under vacuum. Thereafter, cleaned up elute was now washed with high performance liquid chromatography (HPLC) grade methanol. Nitrogen gas streaming was also done in controlled conditions of pressure in petri plates for impurities removal (Ashraf et al., 2022).

A protocol by (Hussain et al., 2020) was followed to determine the imidacloprid, bifenthrin, cypermethrin, chlorpyrifos and deltamethrin contents in dehydrated PP using HPLC system installed with UV–visible detector (PerkinElmer, Series 200, Waltham, MS, USA). Twenty microliter sample was injected into analytical C_18_ column (250 mm × 4.6 mm, I.d.; Supelco, Bellifonte, PA, USA) by an autosampler with Methanol: water (45:55 v/v) as mobile phase. The flow rate of mobile phase was set at 1.5 mL/min with a run time of 20 min for each sample at 254 nm. Standard curves of each pesticide were recorded @ 2, 4, 6, 8 and 10 ppm alongside a reagent blank. Results for pesticide residues were noted in ppm.

### 2.8 Sensory evaluation of microwaved heat-treated PP supplemented unleavened flatbread

From the premix, fifty grams of dough of each treatment was prepared using whole wheat flour (T_0_, control), whole wheat flour supplemented with microwave heat-treated PP @ 2.5, 5, 7.5 and 10 % in clean drinking water. The dough was sheeted into unleavened flatbread and both sides of flatbread were evenly baked at 200 ± 5 °C for 2–3 min on griddle. Raw and microwave heat treated PP supplemented unleavened flatbread were tested for sensory acceptability by a nominated panel of sensory experts (*n* = 15) known for better sensory ability. Sensory experts were already briefed for unbiased assigning of sensory scores for appearance, taste, color, folding ability, texture and overall acceptability of raw and processed unleavened flatbreads in accordance with the 9-Point Hedonic Scale i.e., 1-dislike extremely, to 9-like extremely.

### 2.9 Statistical analysis

Two independent experiments of each analysis were performed. Results for technofunctional properties, physico-chemical composition, mineral profile, antinutrients and extrinsic toxicants i.e., pesticide residues in raw and microwave heat treated PP samples were expressed as means ± standard error (S.E.). Data obtained from the independent experiments of raw and processed PP and supplemented unleavened flatbreads consisting of sensory evaluation study were statistically analyzed via analysis of variance (ANOVA) on the Statistics 8.1 software (Tallahassee, FL) and the results of this section were also presented as means ± standard error (S.E.). Whereas mean values of all analyses were tested for level of significance by least significance difference (LSD) test at 5 % confidence level.

## 3 Result and discussion

### 3.1 Technofunctional properties of raw and processed PP

The data from technofunctional characteristics of dehydrated PP reported significantly (*p* < 0.05) higher magnitudes of rehydration ratio, water solubility index, water absorption index and hygroscopicity i.e., 6.6, 5.5 %, 6.2 g/g and 7.4 in microwave heat processed PP when compared with control and other processing treatments (Table 1). However, the lowest mean concentrations of rehydration ratio, water solubility index, water absorption index and hygroscopicity were observed in acid soaking, acid soaking, alkali soaking and alkali soaking i.e., 5.9, 5.0 %, 5.5 g/g and 6.9, respectively. A retrospective study by (Rodriguez-Sandoval et al., 2012) suggested comparable findings for the water solubility index, water absorption index and swelling power in PP i.e., 7.5 %, 4.5 g/g, and 4.8 %, respectively. However, slight differences in technofunctional characteristics of vegetables powders on processing could be attributed to variation in processing conditions, morphology, cultivars and nutritional composition of vegetables. Presence of higher water absorption index, water solubility index and swelling power in PP could be linked with the phosphate groups repulsion properties of amylopectin, increased hydration ratios on weakening of bonds among crystalline structures.

**Table 1:** Technofunctional properties of raw and processed potato powders.

Treatments	B.D. (g/mL)	R.R.	WSI (%)	WAI (g/g)	S*p* (g/g)	H*g* (%)
**Raw PP**	0.77 ± 0.04^a^	6.40 ± 0.12^ab^	5.21 ± 0.02^b^	5.94 ± 0.03^b^	8.02 ± 0.01^a^	7.14 ± 0.03^c^
**Microwave Heating**	0.73 ± 0.00^b^	6.58 ± 0.01^a^	5.47 ± 0.01^a^	6.15 ± 0.04^a^	7.97 ± 0.01^a^	7.42 ± 0.01^a^
**Blanching**	0.78 ± 0.01^a^	6.00 ± 0.00^c^	5.07 ± 0.02^c^	5.90 ± 0.07^b^	7.83 ± 0.03^b^	7.21 ± 0.00^c^
**Acid Soaking**	0.69 ± 0.00^c^	5.89 ± 0.01^c^	5.00 ± 0.00^c^	5.58 ± 0.01^c^	7.00 ± 0.00^c^	7.32 ± 0.01^b^
**Alkali Soaking**	0.66 ± 0.01^d^	6.15 ± 0.04^bc^	5.05 ± 0.04^c^	5.47 ± 0.01^c^	6.98 ± 0.00^c^	6.92 ± 0.01^d^

Values are expressed as means ± standard error (*n* = 2). Mean values having similar lettering in a column are non–significant at *p* > 0.05; PP = Potato powder.

### 3.2 Nutritional composition of raw and processed PP

Nutritional composition of raw and processed PP portrayed raw PP to exhibit significantly (*p* < 0.05) higher contents of ash, protein and fiber contents i.e., 2.5 %, 10.2 % and 6.3 %, respectively. However, on comparing the treatment efficacies of processing treatments, the data elucidated higher contents of ash, protein and dietary fibers in microwave heat processed PP i.e., 3.1 %, 10 % and 5.7 %, respectively (Table 2). Earlier studies by (Das et al., 2018; Whitney & Simsek, 2020; Yang et al., 2023) indicated the presence of comparable magnitudes of ash, protein and dietary fibers in raw PP ranging between 2.5 and 2.8 %, 7.3–8.3 %, 1.6–11 %, respectively. Likewise, other retrospective studies by (Hidayat, 2019) and (Nascimento & Canteri, 2018) reported the positive correlation of blanching on ash, protein and fiber contents of blanched PP which significantly increased from 3.5 to 7 %, 10.4–11.3 % and 1.3–1.8 %, respectively, however, slight decrease in fat contents of blanched PP from 0.7 to 0.6 % was also observed. The increase in protein contents of blanched PP could be attributed to the ceased catalytic activities of proteases and pectinolytic enzymes in blanched PP. Likewise, slight increment in fiber contents of blanched PP could be attributed to the residues including quinones and melanins produced as a result of polyphenol oxidases and peroxidases actions, which are quantified as insoluble fibers (Nascimento & Canteri, 2018). Another study by (Tian et al., 2016) exhibited significant effect of microwave heat processing on PP ash, protein and fiber contents i.e., 3.8 %, 6.6 % and 9.1 %. However, the same study also portrayed significant impact of blanching on ash protein and fiber contents of PP i.e., 4.1 %, 6.6 % and 5.5 %, respectively. Therefore, potatoes are suggested as plausible source of high quality proteins, inorganic elements and health promoting dietary fibers which are known to combat numerous health disorders such as hypertension, obesity, cardiovascular, diabetes, colon cancer, cerebrovascular disorders, constipation, intestinal problems and micronutrient malnutrition (Osei Tutu et al., 2024; Singh et al., 2023; Yang et al., 2023). On comparing the nutritional potential with the staple cereals, potatoes are known to exhibit lower fats and high-quality proteins (biological value = 0.99), which reveals it as a healthy food for humans (Liang et al., 2019).

**Table 2:** Nutritional composition of raw and processed potato powders (g/100 g).

Treatments	Moisture	Ash	Protein	Fat	Fiber	Carbohydrates	Caloric values (Kcal/100 g)
**Raw PP**	9.38 ± 0.05^a^	2.54 ± 0.01^cd^	10.22 ± 0.47^a^	0.90 ± 0.01^a^	6.26 ± 0.04^a^	70.69 ± 0.37^b^	331. 60 ± 0.28^c^
**Microwave Heating**	8.85 ± 0.04^b^	3.10 ± 0.07^a^	10.00 ± 0.00^ab^	0.82 ± 0.01^b^	5.76 ± 0.18^ab^	71.47 ± 0.14^c^	333.24 ± 0.65^c^
**Blanching**	8.11 ± 0.08^d^	2.73 ± 0.05^bc^	9.72 ± 0.04^ab^	0.63 ± 0.02^c^	5.22 ± 0.16^bc^	73.57 ± 0.25^b^	338.85 ± 0.67^b^
**Acid Soaking**	8.65 ± 0.04^c^	2.44 ± 0.02^d^	8.40 ± 0.28^bc^	0.54 ± 0.01^d^	5.55 ± 0.04^c^	74.42 ± 0.27^b^	337.03 ± 0.18^b^
**Alkali Soaking**	7.60 ± 0.03^e^	2.88 ± 0.01^b^	8.43 ± 0.02^c^	0.47 ± 0.02^d^	4.11 ± 0.08^d^	76.50 ± 0.04^b^	343.98 ± 0.26^a^

Values are expressed as means ± standard error (n = 2). Mean values having similar lettering in a column are non–significant at *p* > 0.05; PP = Potato powder.

### 3.3 Mineral composition of raw and processed PP

The findings on mineral elements of all PP shown significantly higher concentrations of Na, Ca, K, Fe and Zn in raw PP i.e., 50.2 mg/100 g, 62 mg/100 g, 988 mg/100 g, 1.6 mg/100 g and 0.9 mg/100 g, respectively. The Na concentrations varied between 43 and 50 mg/100 g among all thermal and non-thermal processed PP (Table 3). However, Ca and K results showed significantly higher concentration in microwave heat processed and blanched PP i.e., 60.5 mg/100 g and 980.4 mg/100, respectively. Non-significant (*p* < 0.05) effect of processing was recorded in Fe and Zn concentrations of the processed PP. Dietary intake of micronutrients like potassium, sodium and calcium could be helpful in performing several health functions like maintaining the intracellular fluid, osmoregulation, fluid balance, controlling high blood pressure, bone and growth development and supports protein synthesis (Liang et al., 2019). Retrospective studies have validated the presence of Na, Ca, K, Fe and Zn in potato flours from 29 to 35 mg/100 g, 61–71 mg/100 g, 413–1100 mg/100 g, 2.3–2.5 mg/100 g and 2.1–2.2 mg/100 g, respectively (Liang et al., 2019). Another study by (Rahman et al., 2015) portrayed slight decline in K and Ca contents of PP from 413 to 400 mg/100 g and 10–8 mg/100 g, respectively on blanching potatoes for few minutes.

**Table 3:** Mineral contents of raw and processed potato powders (mg/100 g).

Treatments	Na	Ca	K	Fe	Zn
**Raw PP**	50.16 ± 0.59^a^	62.00 ± 0.71^a^	987.80 ± 0.57^a^	1.60 ± 0.04^a^	0.90 ± 0.02^a^
**Microwave Heating**	48.09 ± 0.29^a^	60.50 ± 0.35^ab^	974.50 ± 2.76^bc^	1.53 ± 0.02^a^	0.80 ± 0.02^ab^
**Blanching**	45.17 ± 0.59^b^	57.27 ± 1.22^bc^	980.35 ± 1.87^ab^	1.44 ± 0.03^a^	0.75 ± 0.04^b^
**Acid Soaking**	44.44 ± 0.64^b^	59.50 ± 0.35^ab^	968.00 ± 0.71^c^	1.50 ± 0.07^a^	0.82 ± 0.02^ab^
**Alkali Soaking**	43.17 ± 0.12^b^	55.30 ± 0.21^c^	974.50 ± 0.35^bc^	1.51 ± 0.01^a^	0.72 ± 0.00^b^

Values are expressed as means ± standard error (n = 2). Mean values having similar lettering in a column are non–significant at *p* > 0.05; PP = Potato powder.

### 3.4 Decline in antinutrients in raw and processed PP

**Fig. 1:**
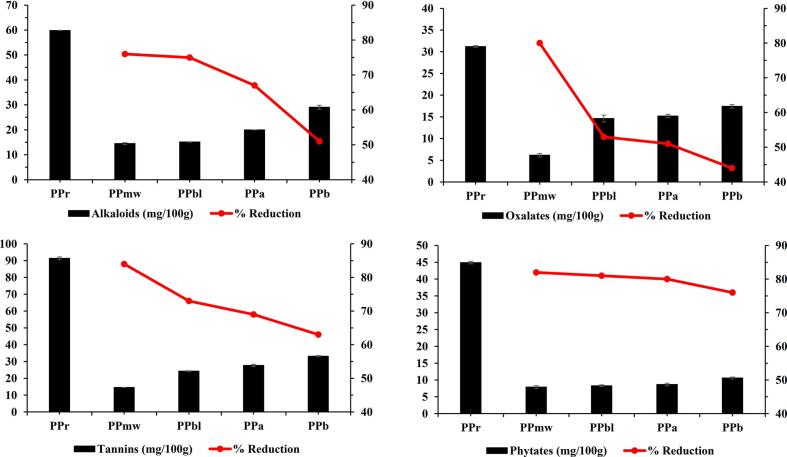
Antinutrients of raw and processed forms of potato powder.

Antinutrients data of PP revealed the highest load of antinutrients consisting of alkaloids, oxalates, tannins and phytates in raw PP i.e., 59.9 mg/100 g, 31.2 mg/100 g, 91.4 mg/100 g and 44.9 mg/100 g, respectively. Alkaloids, oxalates, tannins and phytates contents of processed PP varied between 13.9 and 28.9 mg/100 g, 6.2–17.4 mg/100 g, 14.9–33.2 mg/100 g and 7.9–10.6 mg/100 g, respectively (Fig. 1). Among processing treatments, microwave heat processing of raw PP reported the highest reduction in alkaloids, oxalates, tannins and phytates contents from 60 to 14 mg/100 g (i.e., 77 % reduction), 31–6 mg/100 g (80 % reduction), 91–15 mg/100 g (84 % reduction) and 45–8 mg/100 g (82 % reduction), respectively. Antinutrients are involved in binding of essential minerals i.e., Ca, Zn and Fe and adversely affect absorption and bioavailability. Tannins decrease protein digestibility on intervening with the pH mechanisms and oxalates are linked with formation of kidney stones. Likewise, dietary intake of alkaloids may cause neurological and gastrointestinal disorders (Thakur et al., 2019). An earlier study by (Lakra & Sehgal, 2009) on the antinutrients of PP based food products revealed the presence of higher magnitudes of tannins and phytates i.e., 173 mg/100 g and 139 mg/100 g, respectively. Likewise, another research by (Irakli et al., 2020), depicted the notable effect of microwave heat processing (i.e., 650 W, 2 min) on the rice bran and resulted in the decline of oxalates by 35 %. An earlier investigation by (Gemed, 2014) showed a significant effect of blanching on Anchote tuber flour wherein the results delineated measurable decline in phytates, oxalates and tannins contents by 15 %, 51 % and 41 %, respectively. In another trial by (Thakur et al., 2019), boiling of foods anticipated 20 % reduction in phytates. The decrease in antinutrients like oxalates on soaking, boiling and cooking could be attributed to higher water solubility losses, leaching, lower heat stability and considerable skin rupturing on heat processing, thermal degeneration and complex formation (Thakur et al., 2019). Moreover, available literature has validated the inactivation of phenylalanine-ammonia-lyase enzyme in heat processing of PP which could be collaborated with the reduction of tannins in thermally processed PP (Palharini et al., 2015). Another study by (Waseem et al., 2024) anticipated significant positive correlation of thermal (microwave heat processing and blanching) and non-thermal (acid and base soaking) techniques to ameliorate / decline the antinutrients (oxalates, phytates, tannins and alkaloids) in leafy green vegetables up to 90 %.

### 3.5 Decline in pesticide residues in raw and processed PP

**Fig. 2:**
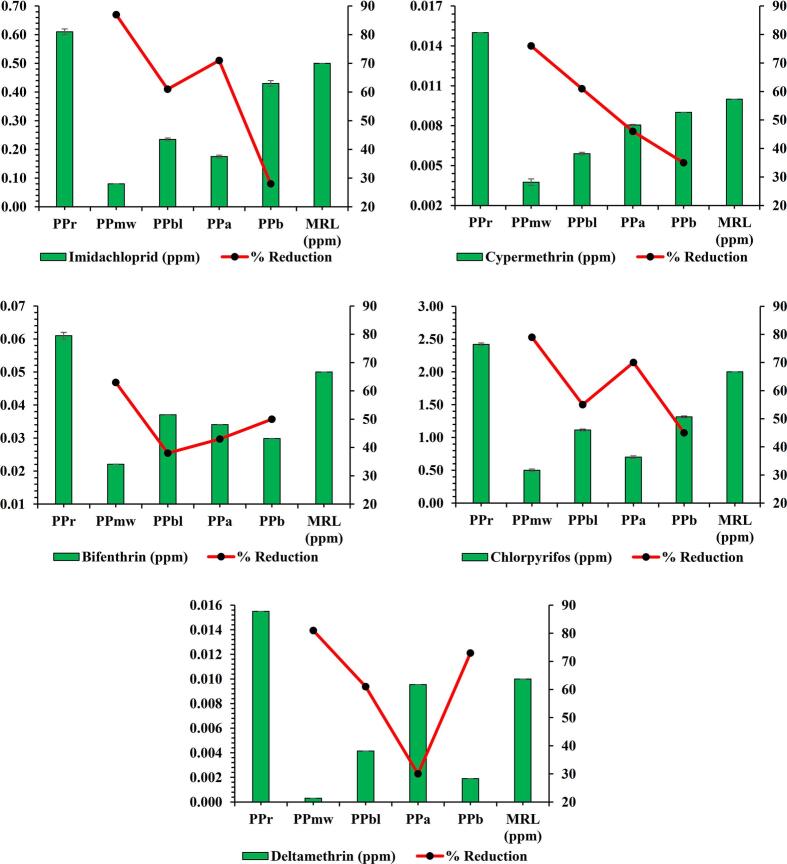
Pesticide residues of raw and processed forms of potato powder.

Results for the residual pesticides of PP portrayed the highest contents of imidacloprid, cypermethrin, bifenthrin, chlorpyrifos and deltamethrin in raw PP i.e., 0.610 ppm, 0.015 ppm, 0.061 ppm, 2.420 ppm and 0.015 ppm, respectively. Contrarily, the residual contents of imidacloprid, cypermethrin, bifenthrin, chlorpyrifos and deltamethrin among processed PP varied between 0.080 and 0.430 ppm, 0.004–0.009 ppm, 0.022–0.037 ppm, 0.500–1.313 ppm, and 0.001–0.009 ppm, respectively (Fig. 2). However, on comparing treatment efficiencies, microwave heat processing reported the highest decline in imidacloprid, cypermethrin, bifenthrin, chlorpyrifos and deltamethrin residues by 87 %, 76 %, 63 %, 79 % and 81 %, respectively. A previous research by (Zhang et al., 2022) elucidated a significant reduction in chlorpyrifos, bifenthrin, malathion, dichlorvos, hexaconazole, kresoxim-methyl, myclobutanil, tebuconazole, triadimefon, cyhalothrin and epoxiconazole contents in fruits and vegetables from 67 to 93 % on microwave heat processing at 700 W for 2 min. A report by (Terfe et al., 2023) showed the washing and boiling to reduce O,P′-DDT pesticide residues by 100 % in potatoes. Also, (Randhawa et al., 2007) portrayed an appreciable reduction in chlorpyriphos contents by 24 % and 53 % on soaking and blanching, respectively. A study of (Kin & Huat, 2010) demonstrated a significant reduction of pesticide residues in potatoes from 44 to 70 %, 30–50 %, and 23–40 % on blanching, alkali soaking and salt soaking, respectively. (Klinhom et al., 2008) showed normal water soaking to alleviate carbaryl and methomyl contents by 88 % and 38 %, respectively. However, the study also delineated the soaking of potatoes in a mixture of salts consisting of NaCl, NaHCO_3_ and acetic acid decreased methomyl contents by 39 %, 43 % and 43 %, respectively while, lowering the forcarbaryl contents by 91 %, 91 % and 90 %, respectively. The reduction in pesticide contents of processed potatoes could be linked to co-evaporation, thermal degradation and leaching losses (Nguyen et al., 2020). Lower efficacy of acid and alkali soaking in eliminating the pesticides could be linked with the higher skin wax, low temperatures, iconic strengths, hydrolytic rates, volatility and water solubility (Hussnain et al., 2021; Yigit & Velioglu, 2020). Earlier data on detoxification of pesticide residues in vegetables have validated the use of thermal and non-thermal detoxification techniques as viable in mitigating the pesticide residues in vegetables ((Waseem et al., 2024).

### 3.6 Sensory qualities of microwave processed potato powder (PPmw) supplemented unleavened flatbreads

**Fig. 3:**
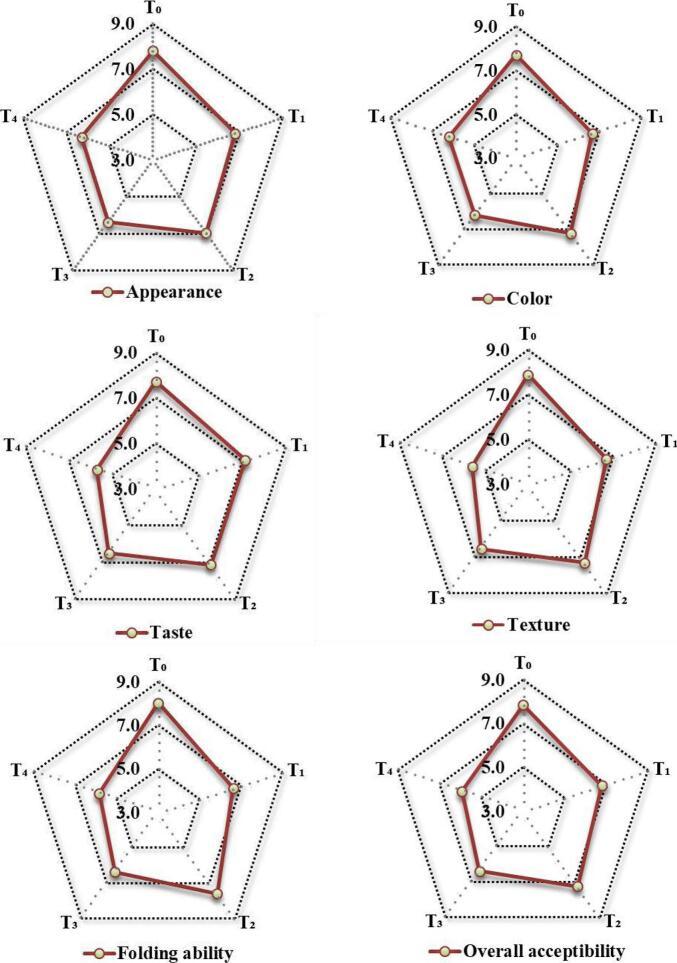
Sensory qualities of microwave processed potato powder (PPmw) supplemented flatbreads.

Sensory evaluation of microwave heat processed PP supplemented unleavened flatbreads indicated variable impact on the sensory traits. The data reported the highest sensory scores for color, taste, texture and overall acceptability for control i.e., 7.7, 7.7, 7.9 and 7.8, respectively. However, among treatment groups the highest sensory scores were assigned to T_2_ (i.e., 5 % supplementation) for color (7.3), taste (7.2), texture (7.3) and overall acceptability (7.3) followed by T_1_ (i.e., 2.5 %), T_3_ (7.5 %), and T_4_ (10 %), respectively (Fig. 3). The sensory scores for texture and folding ability showed non-significant (*p* < 0.05) effect of addition of microwave heat processed PP in the supplemented unleavened flatbreads. The results exhibited the highest sensory acceptability of the finished product ≤5 % supplementation level. Earlier studies by (Das et al., 2018) and (Rahman et al., 2015) have revealed the sensorial acceptability of PP supplemented biscuits <15 % supplementation levels. Likewise, another study by (Chakraborty et al., 2015) depicted better sensory scores for color, flavor and taste of PP supplemented cookies i.e., 6.9, 7.2 and 7.4, respectively and exhibited the best sensory acceptability of the supplemented cookies <10 % replacement. Similarly, a study (Akter et al., 2023) depicted the highest sensory acceptability of dehydrated PP incorporated composite cakes @ 4 % compared with the control. The varied levels of PP sensory acceptability between 4 and 15 % could be attributed to the hydrophilic nature of patatin proteins, weaker gluten networking and hygroscopic nature of PP starches which results in poor sensory scoring of PP value-added food products beyond 15 % supplementation (Jian et al., 2021; Tao et al., 2021). Sensory evaluation results further suggest the use of PP up to 5 % for value addition food products other than baked items like in snack food products, nuggets and confectionery.

## 4 Conclusions

The key results of this study suggest PP as a plausible carrier of health featuring phytonutrients such as high-quality proteins, dietary fibers and essential minerals, which can contribute significant health ameliorating features when consumed in combination or alone. Contrarily, presence of higher levels of antinutrients and pesticides above the allowable limits in tuber crops results in acute to chronic health maladies and nutritional disparities. This study anticipated all the processing techniques to diminish antinutrients and pesticide loads of PP and consequently improved the overall nutrients of all processed PP. Present study suggests a new finding i.e., the microwave heat processing at 1.1 kW for 2 min not merely improved the nutritional status but also resulted in the highest reduction of antinutrients and pesticide residues in potatoes. In conclusion, the study also refers to microwaved heat processed PP as a natural food supplement that is culturally acceptable, sustainable and cheaper source to ameliorate micronutrient inequalities and protein energy malnutrition. Further, the future perspectives of the PP elucidate its utilization in novel food recipes like gravies, soups and curries with improved technofunctional attributes.

## Ethical approval

Informed consent of all participants was obtained before participating in the sensory evaluation of samples. Appropriate protocols for protecting the rights and privacy of all participants were utilized during the execution of the sensory evaluation. All tested samples were safe for consumption. This study is covered by ethical clearance from the Ethics Committee of the College of Basic and Applied Science (ECBAS), University of Ghana, Legon, Accra, Ghana, for the sensory evaluation using trained assessors.

## CRediT authorship contribution statement

**Muhammad Waseem**: Investigation, Formal analysis, Writing – original draft, Methodology. **Saeed Akhtar**: Conceptualization, Project administration, Visualization, Supervision, Resources. **Tariq Ismail**: Conceptualization, Supervision, Visualization, Resources. **Tawfiq Alsulami**: Writing – review & editing, Data curation. **Muhammad Qamar**: Writing – review & editing, Writing – original draft, Validation, Supervision, Software, Methodology, Investigation, Formal analysis. **Dur-e-shahwar Sattar**: Writing – review & editing, Investigation. **Raheel Suleman**: Writing – review & editing, Visualization. **Wisha Saeed**: Writing – review & editing, Methodology, Investigation. **Crossby Osei Tutu**: Writing – review & editing, Validation, Software, Resources, Methodology, Funding acquisition.

## Declaration of competing interest

The authors declare that they have no known competing financial interests or personal relationships that could have appeared to influence the work reported in this paper.

## Data Availability

The data associated with this study are included in article/supp. material/referenced in article.
